# A method to estimate plant density and plant spacing heterogeneity: application to wheat crops

**DOI:** 10.1186/s13007-017-0187-1

**Published:** 2017-05-17

**Authors:** Shouyang Liu, Fred Baret, Denis Allard, Xiuliang Jin, Bruno Andrieu, Philippe Burger, Matthieu Hemmerlé, Alexis Comar

**Affiliations:** 1 INRA, UMR-EMMAH, UMT-CAPTE, UAPV, 228 Route de l’aérodrome CS 40509, 84914 Avignon, France; 2 UMR BioSP, INRA, UAPV, 84914 Avignon, France; 3 UMR ECOSYS, INRA, AgroParisTech, Université Paris-Saclay, 78850 Thiverval-Grignon, France; 4 UMR AGIR, INRA, INPT, 31326 Toulouse, France; 5 Hi-Phen, 84914 Avignon, France

**Keywords:** Wheat, Gamma-count model, Density, RGB imagery, Sampling strategy, Plant spacing heterogeneity

## Abstract

**Background:**

Plant density and its non-uniformity drive the competition among plants as well as with weeds. They need thus to be estimated with small uncertainties accuracy. An optimal sampling method is proposed to estimate the plant density in wheat crops from plant counting and reach a given precision.

**Results:**

Three experiments were conducted in 2014 resulting in 14 plots across varied sowing density, cultivars and environmental conditions. The coordinates of the plants along the row were measured over RGB high resolution images taken from the ground level. Results show that the spacing between consecutive plants along the row direction are independent and follow a gamma distribution under the varied conditions experienced. A gamma count model was then derived to define the optimal sample size required to estimate plant density for a given precision. Results suggest that measuring the length of segments containing 90 plants will achieve a precision better than 10%, independently from the plant density. This approach appears more efficient than the usual method based on fixed length segments where the number of plants are counted: the optimal length for a given precision on the density estimation will depend on the actual plant density. The gamma count model parameters may also be used to quantify the heterogeneity of plant spacing along the row by exploiting the variability between replicated samples. Results show that to achieve a 10% precision on the estimates of the 2 parameters of the gamma model, 200 elementary samples corresponding to the spacing between 2 consecutive plants should be measured.

**Conclusions:**

This method provides an optimal sampling strategy to estimate the plant density and quantify the plant spacing heterogeneity along the row.

## Background

Plant density at emergence is governed by the sowing density and the emergence rate. For a given plant density, the uniformity of plant distribution at emergence may significantly impact the competition among plants as well as with weeds [1, 2]. Plant density and uniformity is therefore a key factor explaining production, although a number of species are able to compensate for low plant densities by a comparatively significant development of individual plants during the growth cycle. For wheat crops which are largely cultivated over the globe, tillering is one of the main mechanisms used by the plant to adapt its development to the available resources that are partly controlled by the number of tillers per unit area. The tillering coefficient therefore appears as an important trait to be measured. It is usually computed as the ratio of the number of tillers per unit area divided by the plant density [3]. Plant density is therefore one of the first variables measured commonly in most agronomical trials.

Crops are generally sown in rows approximately evenly spaced by seedling devices. Precision seedling systems mostly used for crops with plants spaced on the row by more than few centimeters (e.g. maize, sunflower or soybean) distribute seeds relatively evenly along the row. Conversely, for most crops with short distances among plants on the row, e.g. wheat, barley or canola, seeds are distributed non-evenly along the row. This can be attributed both to the mechanisms that free, at a variable frequency, the seed from the seed tank, and the trajectory of the seed that may also vary in the pipe that drives it from the seed tank to the soil. Further, once reaching the soil, the seed may also move with the soil displaced by the sowing elements penetrating the soil surface. Finally, some seeds may abort or some young plants may die because of pests or too extreme local environmental conditions (excess or deficit of moisture, low temperature etc.). The population density and its non-uniformity are therefore recognized as key traits of interests to characterize the canopy at the emergence stage. However, very little work documents the plant distribution pattern along the row, which is partly explained by the lack of dedicated device for accurate plant position measurement [4]. Electromagnetic digitizers are very low throughput and not well adapted to such field measurements [5]. Alternatively, algorithms have been developed to measure the inter-plant spacing along the row for maize crops from top-view RGB (Red Green Blue) images [6, 7]. Improvements were then proposed by using three dimensional sensors [8–10]. However, these algorithms were only validated on maize crops that show relatively simple plant architecture with generally fixed inter-plant spacing along the row.

Manual field counting in wheat crops is still extensively employed as the reference method. Measurements of plant population density should be completed when the majority of plants have just emerged and before the beginning of tillering when individual plants start to be difficult to be identified. Plants are counted over elementary samples corresponding either to a quadrat or to a segment [11]. The elementary samples need to be replicated in the plot to provide a more representative value [12]. For wheat crops, [3] suggested that at least a total of 3 m of rows (0.5 m segment length repeated 6 times) should be counted, while [13] proposed to sample a total of 6 m (segments made of 2 consecutive rows by one meter repeated 3 times in the plot). [14] proposed to repeat at least 4 times the counting in 0.25 m^2^ quadrats corresponding roughly to a total of 6.7 m length of rows (assuming the rows are spaced by 0.15 m). In this case, quadrats may be considered as a set of consecutive row segments with the same length when the quadrat is oriented parallel to the row direction or with variable lengths when the quadrat is oriented differently. Although these recommendations are simple and easy to apply, they may not correspond to an optimal sampling designed to target a given precision level. They may either provide low precision if under sampled or correspond to a waste of human resources in the opposite case.

The sample size required to reach a given precision of the plant density will depend on the population density and the heterogeneity of plant positions along the row that may be described by the distribution of the distances between consecutive plants. This distribution is more likely to be skewed, which could be described by an exponential distribution or a more general one such as the Weibull or the gamma distributions. Fitting such random distribution functions provides not only access to the plant density at the canopy level, but also to its local variation that may impact the development of neighboring plants as discussed earlier.

The objective of this study is to propose an optimal sampling method for plant density estimation and to quantify the heterogeneity of plant spacing along the row. For this purpose, a model is first developed to describe the distribution of the plants along the row. The model is then calibrated over a number of ground experiments. Further, the model is used to compare several plant counting strategies and to evaluate the optimal sampling size to reach a given precision. Finally, the model was also exploited to design a method for quantifying the non-uniformity of plant distribution.

## Methods

### Field experiment

Three sites in France were selected in 2014 (Table 1): Avignon, Toulouse and Grignon. A mechanical seed drill was used in the three sites, which represents the standard practice for wheat crops. In Grignon, five plots were sampled, corresponding to different cultivars with a single sowing density. In Toulouse, five sowing densities were sampled with the same “Apache” cultivar. In Avignon, four sowing densities were sampled also with the same “Apache” cultivar. All measurements were taken at around 1.5 Haun stage [15], when most plants already emerged and were easy to identify visually. This stage is reached approximately 10–14 days after the germination for wheat in France [3]. A total of 14 plots are thus available over the 3 sites showing contrasted conditions in terms of soil, climate, cultivars, sowing density and sowing machine, with however a fixed row spacing of 17.5 cm. All the plots were at least 10 m length and 2 m width.

**Table 1:** The experimental design in 2014 over the three sites

Sites	Latitude	Longitude	Cultivar	Density (seeds m^−2^)
Toulouse	43.5°N	1.5°E	Apache	100, 200, 300, 400, 600
Grignon	48.8°N	1.9°E	Premio; Attlass; Flamenko; Midas; Koréli	150
Avignon	43.9°N	4.8°E	Apache	100, 200, 300, 400

### Image processing

**Fig. 1:**
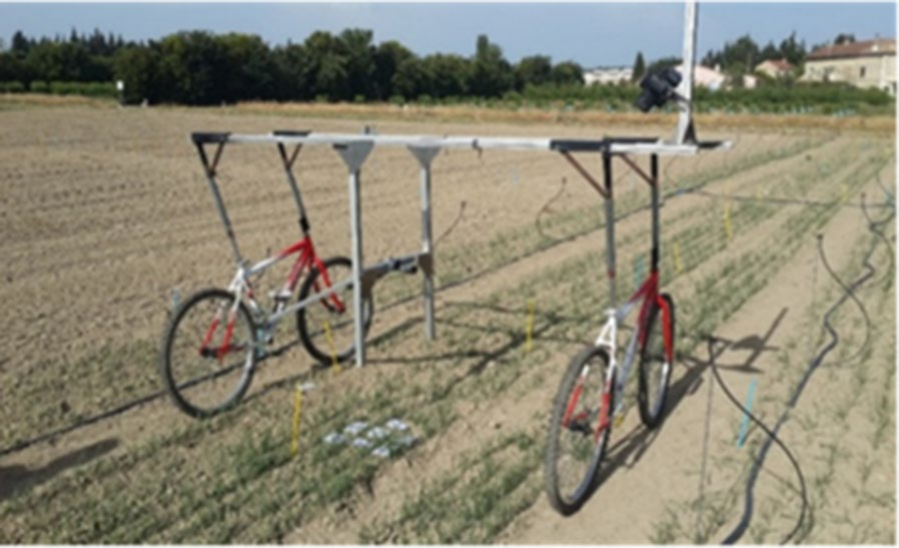
The moving platform used to take the images in the field in 2014

**Fig. 2:**
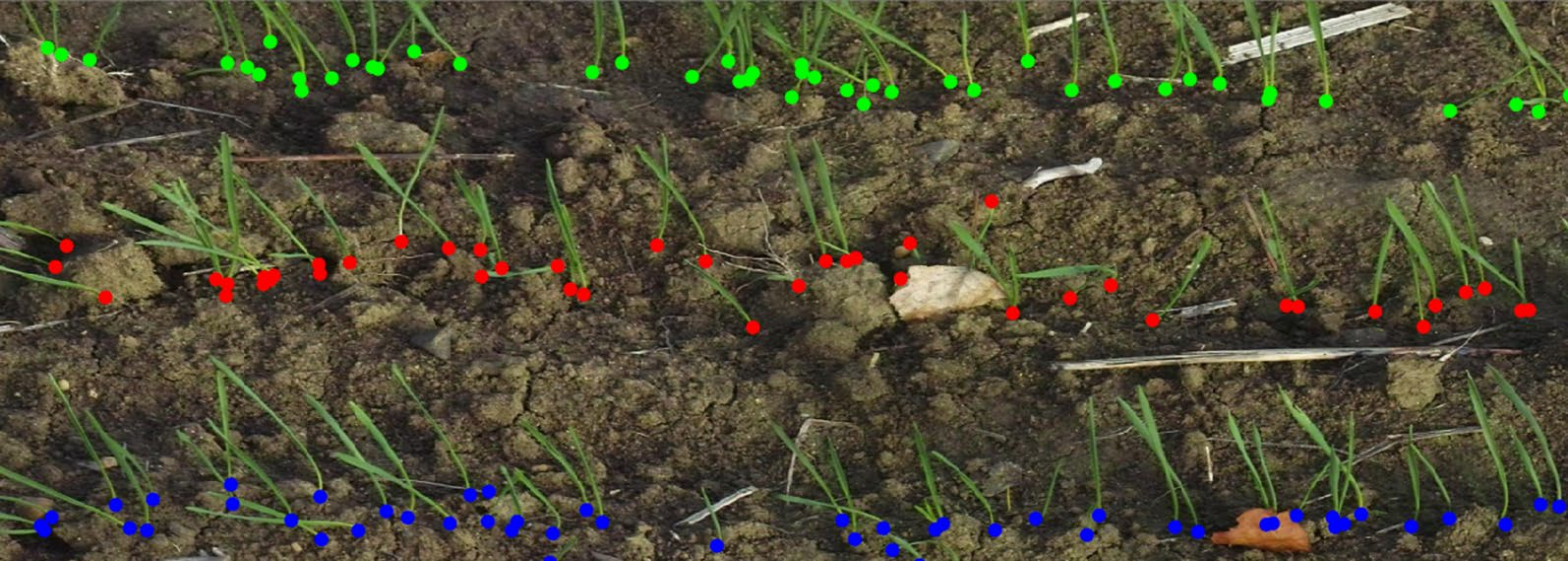
Extraction of plants’ coordinates from the image

A Sigma SD14 RGB camera with a resolution of 4608 by 3072 pixels was installed on a light moving platform (Fig. 1). The camera was oriented at 45° inclination perpendicular to the row direction and was focused on the central row from a distance of about 1.5 m (Fig. 1). The 50 mm focal length allowed to sample about 0.9 m of the row with a resolution at the ground level close to 0.2 mm. Images were acquired along the row with at least 30% overlap to allow stitching. A series of 20 pictures was collected that correspond to three to five rows over about 5 m length. The images were stitched using AutoStitch (http://matthewalunbrown.com/autostitch/autostitch.html) [16]. For each site, one picture was taken over a reference chessboard put on the soil surface to calibrate the image: the transformation matrix derived from the chessboard image was applied to all the images acquired within the same site. It enables to remove perspective effects and to scale the pixels projected on the soil surface. The image correction and processing afterwards was conducted using MATLAB R2016a (code available on request). Coordinates of the plants correspond to the intersection between the bottom of the plant and the soil surface (Fig. 2). They were interactively extracted from the photos displayed on the screen. For each of the 14 plots, the coordinates of at least 150 successive plants from the same row were measured along (X axis) and across (Y axis) of the row. It took between 15 to 30 min to extract the plant coordinates, depending on the density. The precision on the coordinates values along the row is around 1.5 mm as estimated by independent replicates of the process over the same images. Some slightly larger deviations are observed marginally in case of occlusions by stones or straw in the field.

The coordinates ${\text{x}}_{\text{n}}$ of plant *n* (noted ${\text{Plant}}_{\text{n}}$) along the row axis allow to compute the spacing $\Delta {\text{x}}_{\text{n}} = ({\text{x}}_{\text{n}} - {\text{x}}_{{{\text{n}} - 1}} )$ between ${\text{Plant}}_{\text{n}}$ and ${\text{Plant}}_{{{\text{n}} - 1}} .$ The actual plant density expressed in plants per square meter horizontal ground (plants m^−2^) was computed simply as the number of plants counted on the segments, divided by the product of the length of the segments and the row spacing.

### Development and calibration of the plant distribution model

#### Distribution of plant spacing

**Fig. 3:**
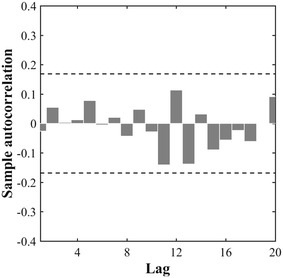
The autocorrelation of the spacing among plants along the row direction illustrated with sowing density of 300 seeds m^−2^ observed over the Toulouse experiment. The lag is expressed as the number of plant spacing between 2 plants along the row direction (X axis). Lags 1–20 are presented. The *upper* and *lower horizontal line* represent the 95% confidence interval around 0

The autocorrelation technique was used to explore the spatial dependency of spacing between successive plants: the linear correlation between $\Delta {\text{x}}_{{{\text{n}} - {\text{m}}}}$ and $\Delta {\text{x}}_{\text{n}}$ where m is the lag is evaluated. Results illustrated in Fig. 3 over the Toulouse site show that the autocorrelation coefficient of inter-plant distance is not significant at 95% confidence interval. The same is observed over the other 13 plots acquired. It is therefore concluded that the positions among plants along the row direction are independent: each observation ∆x could be considered as one independent realization of the random variable ∆X.

**Fig. 4:**
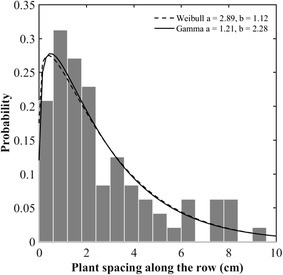
Empirical histogram of the spacing along the row (*gray bars*). The *solid* (respectively *dashed*) *line* represents the fitted gamma (resp. Weibull) distribution. Case of the sowing density 300 seeds m^−2^ observed over the Toulouse experiment. *a* and *b* represent the shape and scale parameters respectively

The distribution of the plant spacing is positively right-skewed (Fig. 4). A simple exponential distribution with only one scale parameter was first tentatively fitted to the data using a maximum likelihood method. However, the Chi square test at the 5% significance level showed that the majority of the 14 plots do not follow this simple exponential distribution law. Weibull and gamma distributions are both a generalization of the exponential distribution requiring an extra shape parameter. Results show that Weibull and gamma distributions describe well (Chi square test at the 5% significance level successful) the empirical distributions over the 14 plots (Fig. 4; Table 2). However, the gamma distribution will be preferred since it provides generally higher *p* value of Chi square test (Table 2) [17]. Besides, the tail of the Weibull distribution tends toward zero less rapidly than that of the gamma distribution: Weibull may show few samples with very large values [18], increasing the risk of overestimation for the larger plant spacing. The gamma distribution was therefore used in the following and writes [19]:

1 $$ {\text{f}}\left( {\Delta {\text{x|a}},{\text{b}}} \right) = \frac{1}{{{\text{b}}^{\text{a}} \varGamma \left( {\text{a}} \right)}}\Delta {\text{x}}^{{{\text{a}} - 1}} {\text{e}}^{{\frac{{ - \Delta {\text{x}}}}{\text{b}}}} \quad \Delta {\text{x}},{\text{a}},{\text{b}} \in {\text{R}}^{ + } $$

where a and b represent the shape and scale parameters respectively. The expectancy ${\text{E}}\left( {\Delta {\text{X}}} \right)$ and variance ${\text{Var}}\left( {\Delta {\text{X}}} \right)$ are simple expressions of the two parameters:

2 $$ {\text{E}}\left( {\Delta {\text{X}}} \right) = {\text{a}} \cdot {\text{b}} $$

3 $$ {\text{Var}}\left( {\Delta {\text{X}}} \right) = {\text{a}} \cdot {\text{b}}^{2} $$

**Table 2:** Parameters of the fitted distributions

Sites	Sowing density (seeds m^−2^)	Cultivar	Gamma			Weibull		
			a	b	p value of Chi square test	a	b	p value of Chi square test
Avignon	100	Apache	1.14	6.38	0.27	7.44	1.07	0.29
	200	Apache	1.25	4.04	0.62	5.29	1.13	0.05
	300	Apache	0.99	2.53	0.38	2.51	1.00	0.56
	400	Apache	0.96	1.50	0.22	1.39	0.94	0.57
Toulouse	100	Apache	1.07	5.01	0.12	5.32	0.99	0.10
	200	Apache	1.39	1.95	0.17	2.86	1.15	0.12
	300	Apache	1.21	2.28	0.94	2.89	1.12	0.94
	400	Apache	1.24	1.37	0.51	1.76	1.10	0.40
	600	Apache	1.16	0.96	0.37	1.14	1.09	0.21
Grignon	150	Premio	1.12	3.37	0.70	3.85	1.06	0.68
	150	Attlass	1.13	2.48	0.69	2.87	1.05	0.67
	150	Flamenko	1.11	3.3	0.92	3.75	1.05	0.92
	150	Midas	1.24	3.03	0.21	3.92	1.12	0.24
	150	Koréli	1.15	2.89	0.24	3.48	1.15	0.18

As a consequence, the coefficient of variation ${\text{CV}}\left( {\Delta {\text{X}}} \right) = \frac{{\sqrt {{\text{var}}\left( {\Delta {\text{X}}} \right)} }}{{{\text{E}}\left( {\Delta {\text{X}}} \right)}}$ is a simple function of the shape parameter:

4 $$ {\text{CV}}\left( {\Delta {\text{X}}} \right) = 1/\sqrt {\text{a}} $$

#### Modeling the distribution of the number of plants per row segment

The plant density evaluated over row segments needs to account for the uncertainties in row spacing. The variability of the row spacing is of the order of 10 mm as reported by [20] which corresponds to CV = 6% using a typical row spacing of 175 mm. For the sake of simplicity, the variability of row spacing will be neglected since it is likely to be small. Further, it is relatively easy to get precise row spacing measurements for each segment and to actually account for the actual row spacing values. Considering a given row spacing, the plant density depends only on the number of plants per unit linear row length. Estimating the number of plants within a row segment is a count data problem analogous to the estimation of the number of events during a specific time interval [19, 21]. Counts are common random variables that are assumed to be non-negative integer or continuous values representing the number of times an event occurs within a given spatial or temporal domain [22]. The gamma-count model suits well our problem with intervals independently following a gamma distribution as in our case. The probability, $\text{P}\left\{ {{\text{N}}_{\text{l}} = {\text{n}}} \right\}$, to get n plants over a segment of length l, writes (Eqs. 5–8 were cited from [19, 21]):

5 $$ {\text{P}}\left\{ {{\text{N}}_{\text{l}} = {\text{n}}} \right\} = \left\{ {\begin{array}{*{20}l} {1 - {\textrm{IG}}\left( {{\text{a}},\frac{l}{b}} \right)} \hfill & {{\text{for}}\;n = 0} \hfill \\ {{\textrm{IG}}\left( {{\text{a}} \cdot {\text{n}},\frac{l}{b}} \right) - {\textrm{IG}}\left( {{\text{a}} \cdot {\text{n}} + {\text{a}},\frac{l}{b}} \right)} \hfill & {{\text{for}}\;n = 1,2, \ldots } \hfill \\ \end{array} } \right. $$

where N_1_ is the number of plants over the segment of length l, and ${\text{IG}}\left( {{\text{a}} \cdot {\text{n}},\frac{l}{b}} \right)$ is the incomplete gamma function:

6 $$ {\text{IG}}\left( {{\text{a}} \cdot {\text{n}},\frac{l}{b}} \right) = \frac{1}{{\varGamma \left( {{\text{a}} \cdot {\text{n}}} \right)}} \int \limits_{0}^{{{\text{l}}/{\text{b}}}} {\text{t}}^{{{\text{a}} \cdot {\text{n}} - 1}} {\text{e}}^{{ - {\text{t}}}} {\text{dt}} $$

where Γ is the gamma Euler function. The expectation and variance of the number of plants over a segment of length l is given by:

7 $$ {\text{E}}\left( {{\text{N}}_{\text{l}} } \right) = \mathop \sum \limits_{{{\text{n}} = 1}}^{\infty } {\text{IG}}\left( {{\text{a}} \cdot {\text{n}},\frac{l}{b}} \right) $$

8 $$ {\text{Var}}\left( {{\text{N}}_{\text{l}} } \right) = \mathop \sum \limits_{{{\text{n}} = 1}}^{\infty } \left( {2{\text{n}} - 1} \right){\text{IG}}\left( {{\text{a}} \cdot {\text{n}},\frac{l}{b}} \right) - \left[ {\mathop \sum \limits_{{{\text{n}} = 1}}^{\infty } {\text{IG}}\left( {{\text{a}} \cdot {\text{n}},\frac{l}{b}} \right)} \right]^{2} $$

Finally, the expectation and variance of the plant density, D_1_, estimated over a segment of length l can be expressed by introducing the row spacing distance, r, assumed to be known:

9 $$ {\text{E}}\left( {{\text{D}}_{\text{l}} } \right) = \frac{{{\text{E}}({\text{N}}_{\text{l}} )}}{{{\text{l}} \cdot {\text{r}}}} $$

10 $$ {\text{Var}}\left( {{\text{D}}_{\text{l}} } \right) =  \frac{{{\text{Var}}({\text{N}}_{\text{l}} )}}{{\left( {{\text{l}} \cdot {\text{r}}} \right)^{2} }} $$

The expectation, ${\text{E}}\left( {{\text{D}}_{\text{l}} } \right)$, converges toward the actual density of the population when 1 → ∞.

The transformed gamma-count model allows evaluating the uncertainty of plant density estimation as a function of the sampling size. The uncertainty can be characterized by the coefficient of variation (CV) as follows:

11 $$ {\text{CV}}\left( {{\text{D}}_{\text{l}} } \right) = \frac{{\sqrt {{\text{Var}}\left( {{\text{D}}_{\text{l}} } \right)} }}{{{\text{E}}\left( {{\text{D}}_{\text{l}} } \right)}} = \frac{{\sqrt {{\text{Var}}\left( {{\text{N}}_{\text{l}} } \right)} }}{{{\text{E}}\left( {{\text{N}}_{\text{l}} } \right)}} $$

**Fig. 5:**
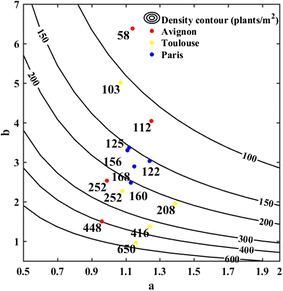
Relationship between parameters *a* and *b* of the gamma-count model for a range of plant density (from Eqs. 6, 7, 9). The *lines* correspond to, 100, 150, 200, 300, 400 and 600 plants m^−2^. The *dots*’ color corresponds to the experimental sites

Several combinations of values of *a* and *b* may lead to the same plant density, but with variations in their distribution along the row (Fig. 5). The fitting of parameters *a* and *b* over the 14 plots using the transformed gamma-count model (Eq. 9) shows that the shape parameter, a, varies from 0.96 to 1.39 and is quite stable. Conversely, the scale parameter, b, appears to vary widely from 0.96 to 6.38, mainly controlling the plant density (Fig. 5). Since the CV depends only on the shape parameter a (Eq. 4), it should not vary much across the 14 plots considered. This was confirmed by applying a one-way analysis of variance on the CV values of the 14 plots available (F = 1.09, P = 0.3685): no significant differences are observed. This result may be partly explained by the fact that the same type of seed drill was used for all the three sites.

## Results

### Optimal sample size to reach a given precision for plant density estimation

The transformed gamma-count model provides a convenient way to investigate the effect of the sampling size on the precision of the density estimates. The precision will be quantified here using the coefficient of variation (CV). The sample size can be expressed either as a given length of the segments where the (variable) number of plants should be counted, or as a (variable) length of the segment to be measured corresponding to a given number of consecutive plants. The two alternative sampling approaches will be termed FLS (Fixed Length of Segments) for the first one, and FNP (Fixed Number of Plants) for the second one.

**Fig. 6:**
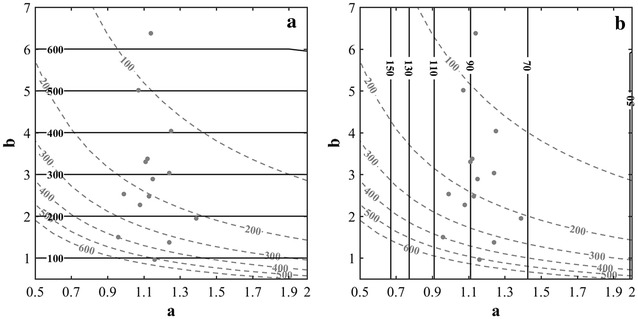
**a** The optimal sampling size length (the *horizontal solid lines*, the length being indicated in cm) used in the FLS approach as a function of parameters *a* and *b* to get CV = 10% for the density estimation. **b** Idem as on the left but the sample is defined by the number of plants to be counted (the *vertical solid lines* with number of plants indicated) for the FNP approach. The *gray dashed lines* correspond to the actual plant density depending also on parameters *a* and *b*. The row spacing is assumed perfectly known and equal to 17.5 cm. The *gray points* represent the 14 plots measured

**Fig. 7:**
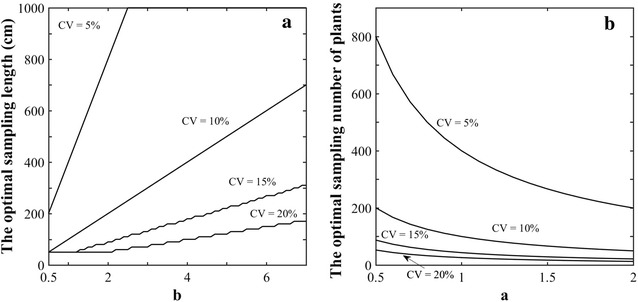
The optimal sampling length for the FLS approach (**a**) and the number of plants for the FNP approach (**b**). The dominant parameter is used (the scale parameter for FLS and the shape coefficient for FNP). The precision is evaluated with the CV = 5, 10, 15 and 20%

When considering the FLS approach, the sample size is defined by the length of segment, *L*, where plants need to be counted. The optimal *L* value for a given target precision quantified by the CV will mainly depend on the current density as demonstrated in Fig. 6a: longer segments are required for the low densities. Conversely, shorter segments are needed for high values of the plant density to reach the same precision. The scale parameter, *b*, that controls the plant density drives therefore the optimal segment length *L* (Fig. 6a). Counting plants over *L* = 5 m (500 cm) provides a precision better than 10% for densities larger than 150 plants·m^−2^ for the most common conditions characterized by a shape coefficient *a* > 0.9. These figures agree well with the usual practice for plant counting as reviewed in the introduction [3, 13, 14]. Increasing the precision quantified by the CV will require longer segments *L* to be sampled (Fig. 7a).

When considering the FNP approach, the sample size is driven by the number, *N*, of consecutive plants that defines to a row segment whose length need to be measured. The simulations of the model (Fig. 6b) show that *N* is mainly independent from the plant density. For the 14 plots considered in this study, segments with 70 < *N* < 110 plants should be measured to reach a precision of CV = 10%. The shape parameter *a* influences dominantly the sample size: more heterogeneous distribution of plants characterized by small values of the shape parameter will require more plants to be counted (Fig. 6b). To increase the precision (lower CV), more plants will also need to be counted (Fig. 7b).

The sampling approach FLS (Fixed Length of segments) is extensively used to estimate the plant density. The 600 cm segment length recommended by [13, 14] agrees well with our results (Figs. 6a, 7a) demonstrating that a precision better than 10% is ensured over large range of densities and non-uniformities. The optimal sampling length (FSL) and optimal number of plants sampled (FNP) was computed for other precision levels for a range of plant densities (Table 3). Results show that the FNP method provides very stable values of the sampling size: it is easy to propose an optimal number of consecutive plants to count to reach a given precision. Conversely, the optimal length of the segment used in the FSL approach varies strongly with the plant density (Table 3): the FLS approach when applied with a segment length chosen a priori without knowing the plant density will result in a variable precision level.

**Table 3:** Optimal sampling size for FSL and FNP over different densities (100, 150, 200, 300, 400 and 600 seeds m^−2^) and precisions (5, 10 and 15%)

Sowing density (seeds m^−2^)	Parameters		CV = 5%		CV = 10%		CV = 15%	
	a	b	FSL (cm)	FNP (Nb. Plt)	FSL (cm)	FNP (Nb. Plt)	FSL (cm)	FNP (Nb. Plt)
100	1.11	5.70	2478	363	620	90	250	39
150	1.15	3.01	1406	348	351	88	130	37
200	1.32	3.00	1398	308	350	78	130	33
300	1.10	2.41	1162	363	291	90	110	39
400	1.10	1.44	774	363	194	90	60	39
600	1.16	0.96	584	348	146	85	50	37

This was calculated using the average values of the parameters a and b of the gamma distribution derived for each density over the 14 plots available

### Sampling strategy to quantify plant spacing variability on the row

The previous sections demonstrated that the scale and shape parameters could be estimated from the observed distribution of the plant spacing. However, the measurement of individual plant spacing is tedious and prone to errors as outlined earlier. The estimation of these parameters from the variability observed between small row segments containing a fixed number of plants will therefore be investigated here. This FNP approach is preferred here to the FSL one because there will be no additional uncertainties introduced by the position of the first and last plants of the segment with the corresponding start and end of the segment. These uncertainties may be significant in case of small segments in the FLS approach.

The probability distribution of a gamma distribution can be expressed as the sum of an arbitrary number of independent individual gamma distributions [23]. This property allows to compute the distribution of a segment of length L_n_ corresponding to *n* plant spacing between (*n* + 1) consecutive plants with ${\text{L}}_{\text{n}} = \sum \nolimits_{{{\text{i}} = 1}}^{\text{n}} \Delta {\text{x}}_{\text{i}} ,$ as a gamma distribution with *n* · *a* as shape parameter and the same scale parameter *b* as the one describing the distribution of ∆X.

12 $$ {\text{L}}_{\text{n}} \sim{\text{Gamma}}\left( {{\text{n}} \cdot a,b} \right) $$

The parameters *a* and *b* will therefore be estimated by adjusting the gamma model described in Eq. 12 for the given value of *n* + 1 consecutive plants.

**Fig. 8:**
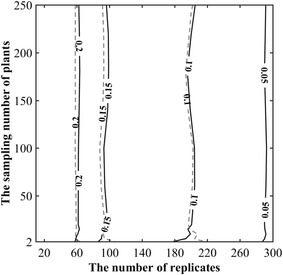
Contour plot of the CV associated to the estimates of parameters *a* (*solid line*) and *b* (*dashed line*) as a function of the number of replicates of individual samples made of n plants (the *y* axis). The *solid* (respectively *dashed*) isolines correspond to the CV of parameter a (respectively parameter *b*). These simulations were conducted with [a, b] = [1.10, 2.27]

The effect of the sampling size on the precision of *a* and *b* parameters estimation was further investigated. A numerical experiment based on a Monte-Carlo approach was conducted considering a standard case corresponding to the average of the 14 plots sampled in 2014 with *a* = 1.10 and *b* = 2.27. The sampling size is defined by the number of consecutive plants for the FNP approach considered here and by the number of replicates. For each sampling size 300 samples were generated by randomly drawing in the gamma distribution (Eq. 12) and parameters *a* and *b* were estimated. The standard deviation between the 300 estimates of *a* and *b* parameters was finally used to compute the corresponding CV. This process was applied to a number of replicates varying between 20 to 300 by steps of 10 and a number of plants per segment varying between 2 (i.e. spacing between two consecutive plants) to 250 within 12 steps. This allows describing the variation of the coefficient of estimated values of parameters *a* and *b* as a function of the number of replicates and the number of plants (Fig. 8).

Results show that the sensitivity of the CV of estimates of parameters *a* and *b* are very similar (Fig. 8). The sensitivity of parameters *a* and *b* is dominated by the number of replicates: very little variation of CV is observed when the number of plants per segment varies (Fig. 8). Parameters *a* and *b* require about 200 replicates independently from the number of plants per segment. It seems therefore more interesting to make very small segments to decrease the total number of plants to count.

Additional investigations not shown here for the sake of brevity, confirmed the independency of the number of replicates to the number of plants per segment when parameters *a* and *b* are varying. Further, the number of replicates need to be increased as expected when the shape parameter *a* decreases (i.e. when the plant spacing is more variable) to keep the same precision on estimates of *a* and *b* parameters.

## Discussion and conclusions

A method was proposed to estimate plant density and sowing pattern from high resolution RGB images taken from the ground. The method appears to be much more comfortable as compared with the standard outdoor methods based on plant counting in the field. Images should ideally be taken around Haun stage 1.5 for wheat crops when most plants have already emerged and tillering has not yet started. Great attention should be paid to the geometric correction in order to get accurate ortho-images where distances can be measured accurately. The processing of images here was automatic except the last step corresponding to the interactive visual extraction of the plants’ coordinates in the image. However, recent work [24, 25] suggests that it will be possible to automatize this last step to get a fully high-throughput method.

The method proposed is based on the modeling of the plant distribution along the row. It was first demonstrated that the plant spacing between consecutive plants are independent which corresponds to a very useful simplifying assumption. The distribution of plant spacing was then proved to follow a gamma distribution. Although the Weibull distribution showed similar good performance, it was not selected because of the comparatively heavier tails of the distribution that may create artefacts. Further the Weibull model does not allow to simply derive the distribution law of the length of segments containing several consecutive plants [26]. The gamma model needs a scale parameter that drives mostly the intensity of the process, i.e. the plant density, and a shape parameter that governs the heterogeneity of plant spacing. This model was transformed into a count data model to investigate the optimal sampling required to get an estimate of plant density for a given precision level.

**Fig. 9:**
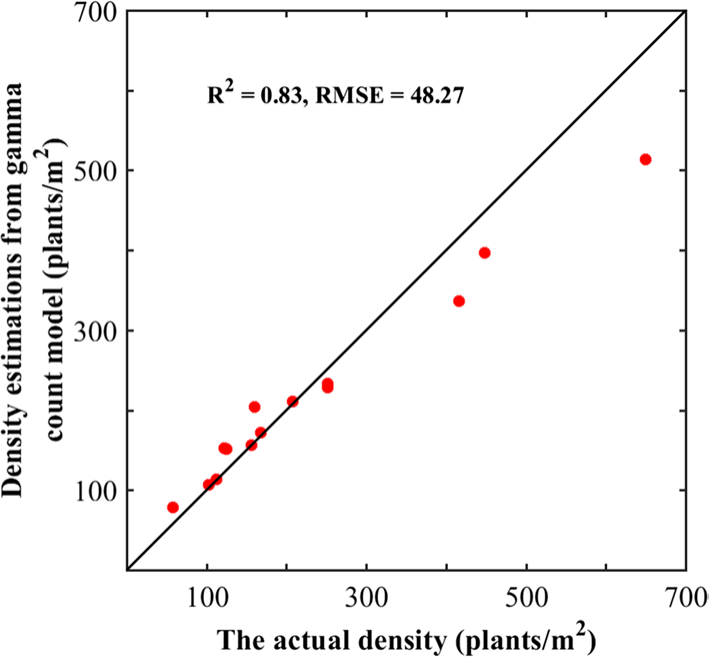
Comparison between the actual density and that estimated from the gamma-count model

The adjustment of the gamma-count model on the measured plant spacing using a maximum likelihood method provides an estimate of the plant density (Eq. 9). The comparison to the actual plant density (Fig. 9) simply computed as the number of plants per segment divided by the area of the segments (segment length by row spacing), shows a good agreement, with RMSE ≈ 50 plants m^−2^ over the 14 plots available. The model performs better for the low density with a RMSE of 21 plants m^−2^ for density lower than 400 plants m^−2^. These discrepancies may be mainly explained by the accuracy in the measurement of the position of individual plants (around 1–2 mm). Uncertainties on individual plant spacing will be high in relative values as compared to that associated with the measurement of the length of the segment used in the simple method to get the ‘reference’ plant density. Hence it is obviously even more difficult to get a good accuracy in plant spacing measurements for high density, i.e. with a small distance among plants. In addition, small deviations from the gamma-count model are still possible, although the previous results were showing very good performance.

The model proposed here concerns mainly relatively nominal sowing, i.e. when the sowing was successful on average on the row segments considered: portions of rows with no plants due to sowing problems or local damaging conditions (pests, temperature and moisture). The sowing was considered as nominal on most of the plots investigated in this study, with no obvious ‘accidents’. However, it is possible to automatically identify from the images the unusual row segments with missing plants or excessive concentration of plants [25]. Rather than describing blindly the bulk plant density, it would be then preferred to get a nested sampling strategy: the unusual segments could be mapped extensively, and the plant density of nominal and unusual segments could be described separately using the optimal sampling proposed here.

This study investigated the sampling strategy to estimate the plant density with emphasis on the variability of plant spacing along the row, corresponding to the sampling error. However additional sources of error should be accounted for including measurement biases, uncertainties in row spacing or non-randomness in the sample selection [27– 29]. Unlike sampling error, it could not be minimized by increasing sampling size. The non-sampling error may be reduced by combining a random sampling selection procedure with a measurement method ensuring high accuracy including accounting for the actual values of the row spacing measured over each segment [30].

Optimal sampling requires a tradeoff between minimum sampling error obtained with maximum sampling size and minimum cost obtained with minimum sampling size [31]. The optimal sampling strategy should first be designed according to the precision targeted here quantified by the coefficient of variation (CV) characterizing the relative variability of the estimated plant density between several replicates of the sampling procedure. The term ‘optimal’ should therefore be understood as the minimum sampling effort to be spent to achieve the targeted precision. Two approaches were proposed: the first one considers a fixed segment length (FSL) over which the plants have to be counted; the second one considers a fixed number of successive plants (FNP) defining a row segment, the length of which needs to be measured. The first method (FLS) is the one generally applied within most field experiments. However, we demonstrated that it is generally sub-optimal: since the segment length required to achieve a given CV depends mainly on the actual plant density: the sampling will be either too large for the targeted precision, or conversely too small, leading to possible degradation of the precision of plant density estimates. Nevertheless, for the plant density (>100 plants m^−2^) and shape parameter (*a* > 0.9) usually experienced, a segment length of 6 m will ensure a precision better than 10%. The second approach (FNP) appears generally more optimal: it aims at measuring the length of the segment corresponding to a number of consecutive plants that will depend mainly on the targeted precision. Results demonstrate that in our conditions, the density should be evaluated over segments containing 90 plants to achieve a 10% precision. The sampling size will always be close to optimal as compared to the first approach where optimality requires the knowledge of the plant density that is to be estimated. Further, the FNP approach is probably more easy to implement with higher reliability: as a matter of facts, measuring the length of a segment defined by plants at its two extremities is easier than counting the number of plants in a fixed length segment, where the extremities could be in the vicinity of a plant and its inclusion or not in the counting could be prone to interpretation biases by the operator. The total number of plants required in a segment could be split into subsamples containing smaller number of plants that will be replicated to get the total number of plants targeted. This will improve the spatial representativeness. Overall, the method proposed meets the requirements defined by [32, 33] for the next genearation of phenotyping tools: increase the accuracy, the precision and the throughput while reducing the labor and budgetary costs.

The gamma-count model proved to be well suited to describe the plant spacing distribution along the row over our contrasted experimental situations. It can thus be used to describe the heterogeneity of plant spacing as suggested by [20]. This may be applied for detailed canopy architecture studies or to quantify the impact of the sowing pattern heterogeneity on inter-plant competition [1, 2]. The heterogeneity of plant spacing may be described by the scale and shape parameters of the gamma model. Quantification of the heterogeneity of plant spacing requires repeated measurements over segments defined by a fixed number of plants. Our results clearly show that the precision on estimates of the gamma count parameters depends only marginally on the number of plants in each segment. Conversely, it depends mainly on the number of segments (replicates) to be measured. For the standard conditions experienced in this study, the optimal sampling strategy to get a CV lower than 10% on the two parameters of the gamma distribution would be to repeat 200 times the measurement of plant spacing between 2 consecutive plants.
